# Neonatal cytogenetic validation demonstrates high accuracy of single-nucleotide polymorphism-based non-invasive prenatal testing: a 4466-case single-center study

**DOI:** 10.1038/s10038-026-01483-w

**Published:** 2026-06-04

**Authors:** Shiho Uchida, Yuki Mizuguchi, Suguru Sato, Kou Sueoka, Mamoru Tanaka

**Affiliations:** 1 Department of Obstetrics and Gynecology, Keio University School of Medicine, Shinjuku-ku, Tokyo Japan; 2 Rose Ladies Clinic, Setagaya-ku, Tokyo Japan; 3 Graduate School of Public Health, Shizuoka Graduate University of Public Health, Shizuoka City, Shizuoka Japan; 4 Fujisawa IVF Clinic, Fujisawa City, Kanagawa Japan

**Keywords:** Genetics research, Outcomes research

## Abstract

Non-invasive prenatal testing (NIPT) is widely used for fetal aneuploidy screening; however, most evaluations of test accuracy rely on pregnancy outcomes rather than cytogenetic confirmation after birth, and evidence regarding the real-world performance of single-nucleotide polymorphism (SNP)-based NIPT remains limited. We analyzed prospectively collected clinical data from pregnant women at high risk of fetal aneuploidy who underwent SNP-based NIPT between 2013 and 2022 to evaluate its analytical accuracy using neonatal buccal mucosal fluorescence in situ hybridization (FISH). Positive NIPT results were followed by amniocentesis, whereas negative results were systematically validated by postnatal buccal mucosal FISH. Sensitivity, specificity, positive predictive value (PPV), and negative predictive value (NPV) were calculated for trisomies 13, 18, and 21. Among 4466 women undergoing initial testing, a total of 4502 tests were performed, including 36 repeat tests after redraw. Of these tests, 4366 (97.0%) were negative, 73 (1.6%) were positive, and four (0.1%) yielded “no call” results after redraw. Sensitivity for trisomies 13, 18, and 21 was 94.6%, 100.0%, and 80.0%, respectively, with a specificity of 99.9% for all three conditions. The overall PPV was 89.1% and the NPV was 99.9%. Two false-negative cases (0.06%) were identified, both attributable to low-level mosaicism. Of the initial “no call” cases, 88.9% yielded reportable results after redraw. These findings demonstrate that SNP-based NIPT shows high analytical performance when validated with neonatal cytogenetic confirmation, while rare false-negative results due to mosaicism highlight the biological limitations of cfDNA-based screening and the importance of cytogenetic confirmation when ultrasound abnormalities are present.

## Introduction

Since Lo et al. first reported in 1997 that cell-free DNA (cfDNA) of fetal origin is present in maternal plasma [1], prenatal diagnosis using cfDNA has progressively advanced. In 2008, Chiu et al. demonstrated that fetal trisomy 21 could be detected non-invasively by applying massively parallel sequencing (MPS) to cfDNA in maternal plasma, establishing the basis for the counting-based approach to non-invasive prenatal testing (NIPT) [2]. These technological advances led to the clinical implementation of NIPT for common autosomal trisomies in many countries.

In Japan, NIPT was introduced in 2013 as clinical research for pregnant women at high risk of carrying a fetus with chromosomal aneuploidy. In 2020, the American College of Obstetricians and Gynecologists (ACOG) and the Society for Maternal-Fetal Medicine (SMFM) published a practice bulletin stating that all pregnant women, regardless of risk, should receive information about available screening and cytogenetic confirmation and make an informed choice. In Japan, national guidelines were revised in 2022 to expand access to accredited facilities for NIPT rather than to broaden clinical indications [3], and the number of tests is expected to increase.

NIPT has been reported to have a high level of accuracy compared with maternal serum marker tests and ultrasound examinations [4], but its accuracy has generally been evaluated based on pregnancy outcomes rather than cytogenetic confirmation using samples obtained directly from newborns. This has been consistently cited as a major limitation in previous studies [5, 6].

Currently, two analytical approaches are used in NIPT: the counting method (MPS method) and the single-nucleotide polymorphisms (SNP) method. Unlike MPS method that relies on quantitative differences in chromosomal representation, SNP method employs haplotype-based analysis of SNPs. This enables estimation of fetal fraction and improved discrimination between maternal and fetal cfDNA signals, allowing detection of biological complexities such as triploidy and vanishing twin pregnancies. In Japan, the MPS method has been the standard approach since the introduction of NIPT in Japan [7], and there are no reports on the accuracy of SNP-based NIPT in Japan that have been systematically validated using cytogenetic confirmation.

The present study has two unique strengths. First, we evaluated the performance of SNP-based NIPT, which has not been previously assessed in Japan. Second, we performed cytogenetic confirmation not only in positive cases but also across a large cohort of negative cases using postnatal buccal mucosal fluorescence in situ hybridization (FISH) analysis. By leveraging these strengths, we examined the true analytical accuracy of SNP-based NIPT at a single-center over a 10-year period using neonatal cytogenetic confirmation.

## Materials and methods

### Study population

The study population consisted of pregnant women at high risk of carrying a fetus with chromosomal aneuploidy. High-risk status was defined by the presence of one or more of the following conditions: (1) fetal ultrasound examination indicating structural abnormalities (findings suggestive of chromosomal aneuploidy, including increased nuchal translucency (NT) and cystic hygroma), (2) a positive maternal serum marker test, (3) a previous pregnancy resulting in a child with chromosomal aneuploidy, (4) advanced maternal age (≥35 years), and (5) an increased risk of fetal trisomy 21 or 13 due to a Robertsonian translocation in either parent.

Multiple pregnancies, including recognized vanishing twins, were excluded from this study because such conditions may affect cfDNA composition and the interpretation of NIPT results.

### Study design

This study was conducted as a clinical research program following the “Opinion on Genetic Testing and Diagnosis Performed Prenatally” issued by the Japan Society of Obstetrics and Gynecology (JSOG) in June 2013 [8], and was a single-center observational cohort study. Pregnant women who underwent SNP-based NIPT at our institution were prospectively enrolled. Clinical follow-up, including prenatal care and delivery, was not necessarily performed at our institution. However, pregnancy outcomes were ascertained through medical records and/or postnatal follow-up.

The analytical performance of NIPT was evaluated by comparing test results with cytogenetic outcomes. In cases without invasive diagnostic testing, postnatal confirmation was performed using FISH analysis of neonatal buccal mucosal cells. The present analysis represents a retrospective evaluation of prospectively collected clinical data.

The FISH analysis was performed as part of a research validation protocol with no additional cost to participants, and without involvement of the testing entity in study design, analysis, or interpretation.

### Outcomes

The primary outcome was the analytical accuracy of SNP-based NIPT, including sensitivity, specificity, positive predictive value (PPV), and negative predictive value (NPV) for trisomies 13, 18, and 21.

Secondary outcomes included the annual number of examinations, maternal age, gestational age at testing, indication for testing, BMI, fetal fraction rate (FFR), pregnancy outcomes, and the distribution of PPV/NPV.

### Genetic counseling and NIPT procedure

Genetic counseling was provided to pregnant women and their partners who wished to undergo testing from 10 weeks of gestation. Prior to testing, all participants were asked to view a standardized educational video of approximately 30 minutes, which explained the principles, scope, limitations, and possible outcomes of NIPT. This was followed by face-to-face counseling, provided by a multidisciplinary team consisting of obstetricians, clinical geneticists, and certified genetic counselors. Counseling was individualized according to each case for approximately 15 min per couple (or more if necessary).

During this session, we explained that prenatal testing aims to evaluate fetal conditions as accurately as possible and to support informed decision-making regarding pregnancy management and future family planning for the pregnant woman and her partner. The purpose of this counseling framework was to support pregnant women and their partners in making informed decisions regarding prenatal testing.

In addition, participants received explanations regarding the indications for NIPT, its limitations, the handling of test results, and the policy for result disclosure, and they were given opportunities to ask questions. Especially, in cases with fetal ultrasound findings suggestive of chromosomal aneuploidy, including increased NT, additional explanation was provided regarding the heterogeneous etiology of such findings and the limitations of NIPT in detecting all underlying conditions, as well as the option of invasive diagnostic testing. After receiving genetic counseling, some individuals chose not to undergo NIPT, while others elected to proceed directly with invasive diagnostic testing such as amniocentesis. If a decision regarding testing could not be reached during a single visit, couples were encouraged to return for additional counseling on a separate day, thereby supporting autonomous and fully informed decision-making.

Although SNP-based NIPT has the technical capability to detect smaller copy number variations, participants were informed that, in accordance with the 2013 statement of JSOG, the analytical scope of this study was limited to trisomies 13, 18, and 21. It was also explained that incidental findings, including microdeletions, duplications, and other genetic abnormalities, were not included in the analytical scope and would therefore not be disclosed.

During the study period, the increasing availability of NIPT at unlicensed facilities led to a rise in inquiries regarding additional testing options, such as sex chromosome analysis and genome-wide screening. In response, the educational video and counseling materials were revised to provide clearer explanations regarding the clinical and ethical rationale for restricting the scope of testing to the three trisomies in certified settings. Through these efforts, we aimed to enhance participant understanding and support appropriate informed decision-making.

When a participant wished to undergo the test, written informed consent was obtained from all participants. Approximately 20 mL of blood was collected from the pregnant women. All cfDNA samples were processed at a CLIA- and CAP-certified laboratory (Natera Inc., San Carlos, CA, USA). Neonatal buccal mucosal FISH analysis was performed at FLSC Inc. (Nagoya, Japan). The test was performed using a validated methodology that included the isolation of cfDNA, polymerase chain reaction amplification targeting 19,488 SNPs, high-throughput sequencing, and analysis using a proprietary haplotype-based algorithm developed by Natera Inc. (Next-generation Aneuploidy Testing Using SNPs: NATUS algorithm) [9–11]. The report included a risk score for each of the three aneuploidies, and the risk score was calculated by combining the maximum likelihood estimate calculated by the NATUS algorithm with the prior risk based on maternal age and gestational age. All samples with a risk score of 1/100 or higher were reported as having a high risk of fetal aneuploidy, and samples with a risk score of less than 1/100 were judged to be negative. In cases where the cfDNA concentration in the blood was lower than the reference value, or where the data did not meet quality-control metrics, the result was judged to be “no call”.

### Follow-up system and validation

If the result was positive, diagnostic testing was performed using amniocentesis. If the test result was negative, or if the patient did not wish to confirm the diagnosis even if the result was positive, a diagnostic confirmation was performed by neonatal buccal mucosal FISH analysis of the chromosomes 13, 18, and 21. In addition, if a miscarriage or stillbirth occurred during the course of the pregnancy, chorionic tissue was collected from the stillborn tissue and subjected to FISH analysis.

The samples were classified into the following categories.

The definitions of the terms are as follows:

1. true positive (TP): positive NIPT with trisomy on cytogenetic confirmation;
2. false positive (FP): positive NIPT without trisomy on cytogenetic confirmation;
3. true negative (TN): negative NIPT without trisomy on cytogenetic confirmation;
4. false negative (FN): negative NIPT with trisomy on cytogenetic confirmation;
5. “Insufficient information” refers to cases that do not match any of the above.

The five categories were determined based on the results of diagnostic testing or FISH analysis of the newborn, and not on the physical findings of the newborn or the findings of ultrasound examinations during pregnancy. Cases of mosaicism were classified as cases of aneuploidy. Cases without cytogenetic confirmation were excluded from accuracy calculations.

The sensitivity, specificity, PPV, and NPV were calculated using the following formulas

$${{{\rm{Sensitivity}}}} 	 =[{{{\rm{TP}}}}]/[{{{\rm{TP}}}}+{{{\rm{FN}}}}],{{{\rm{Specificity}}}}=[{{{\rm{TN}}}}]/[{{{\rm{TN}}}}+{{{\rm{FP}}}}],{{{\rm{PPV}}}} \\ 	 =[{{{\rm{TP}}}}]/[{{{\rm{TP}}}}+{{{\rm{FP}}}}],{{{\rm{NPV}}}}=[{{{\rm{TN}}}}]/[{{{\rm{TN}}}}+{{{\rm{FN}}}}]$$

## Results

### Overview of the test (Fig. 1)

From October 2013 to June 2022, 4466 women underwent NIPT. The number of tests peaked at 974 in 2015 and declined thereafter. The average maternal age at the time of testing was 38.3 years (range 25–47 years), and 94.3% of the patients were aged ≥35 years. The average gestational age at the time of testing was 12.8 weeks (range 10–18 weeks). The main reason for the test was advanced maternal age (94.5%), followed by a history of chromosomally abnormal pregnancy (2.8%) and structural abnormalities detected on ultrasound (2.4%). Information on the mode of conception was partially available for cases from November 2017 onward. Among 1557 cases during this period, 540 pregnancies (34.7%) were conceived using assisted reproductive technology. Information regarding preimplantation genetic testing was not available. Approximately 89.1% of the participants received prenatal care at external institutions.

### NIPT results (Fig. 2)

In the first-draw NIPT, 4339 of the 4466 tests (97.2%) were negative, 68 (1.5%) were positive, and 59 (1.3%) yielded “no call” results. Of the 59 “no call” cases, 23 (23/59, 39.0%) proceeded directly to amniocentesis or miscarried before repeat sampling, while the remaining 36 (61.0%) underwent a redraw. Among the 36 redraws, 27 (75.0%) were negative, five (13.9%) were positive, and four (11.1%) remained “no call”, resulting in 32 reportable results (88.9%). After including redraw results, the final distribution was 4366 negative (97.0%), 73 positive (1.6%), and four “no call” cases (0.1%).

**Fig. 1:**
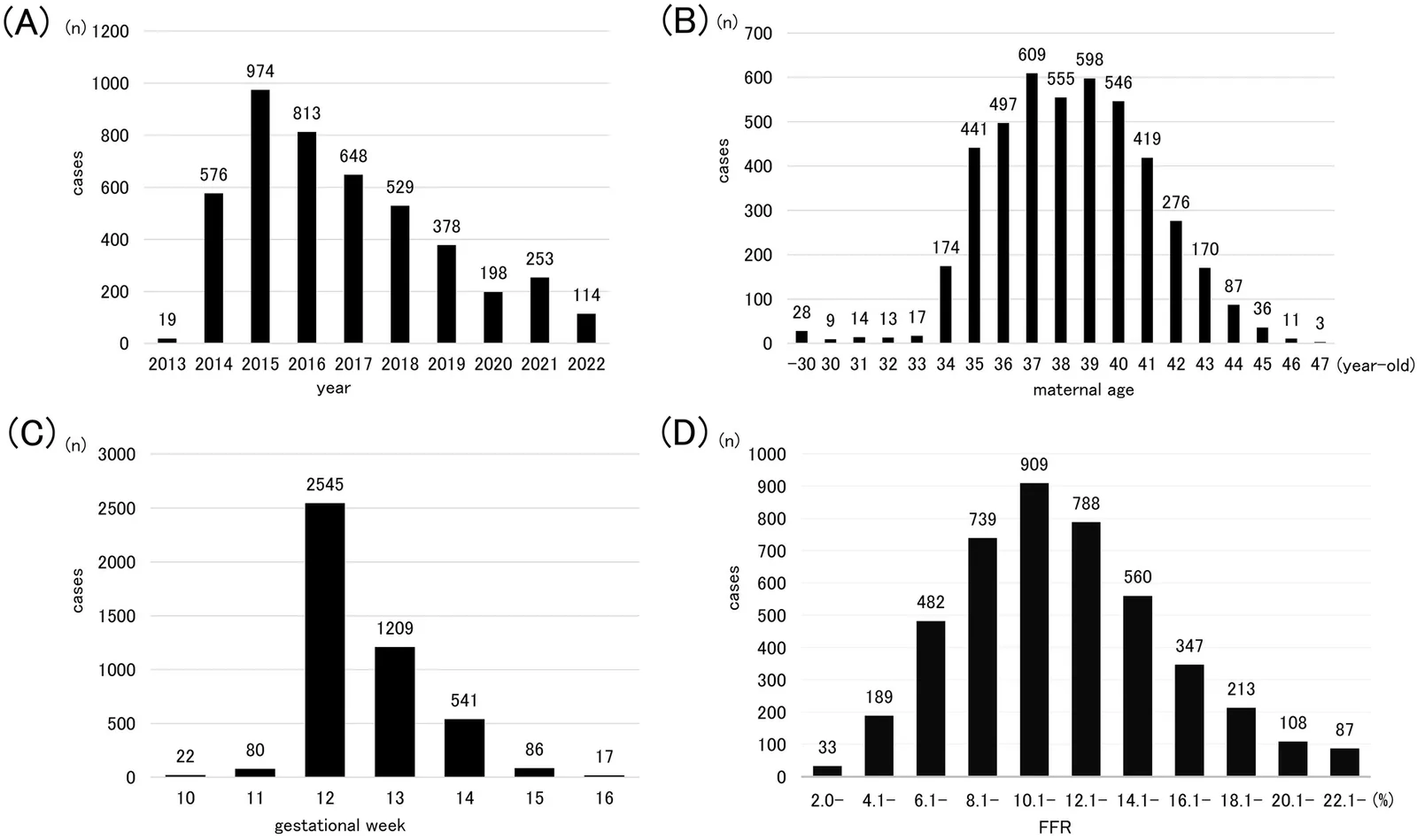
Annual number of SNP-based NIPT tests and patient characteristics at the time of testing. **A** Annual number of SNP-based NIPT tests performed from 2013 to 2022. **B** Distribution of maternal age at the time of testing. **C** Distribution of gestational age at the time of testing. **D** Distribution of fetal fraction rate (FFR) among all SNP-based NIPT samples

The mean FFR was 12.2% (range 2.2–38.1%), with 98.4% of tests showing an FFR ≥ 4%, 0.7% <4%, and 0.9% with no measurable FFR (Fig. 1).

**Table 1:** Positive NIPT results and cytogenetic confirmation

	Number of screened “positive”	TP	FP	PPVs
Trisomy 21	37	35	2	94.6%
Trisomy 18	20	18	2	90.0%
Trisomy 13	7	4	3	57.1%
Total	64	57	7	89.1%

*TP* true positive, *FP* false positive, *PPV* positive predictive value

### Positive cases and PPV (Table 1)

Of the 73 patients with positive NIPT results, nine miscarried before karyotype confirmation, and the remaining 64 underwent cytogenetic confirmation. Therefore, PPV was calculated among cases with definitive cytogenetic confirmation. Seven FP cases were identified, yielding an overall PPV of 89.1% (57/64), and PPV for trisomy 13, 18, and 21 were 57.1% (4/7), 90.0% (18/20), and 94.6% (35/37), respectively.

### FN cases (Fig. 3)

Postnatal cytogenetic confirmation using neonatal buccal mucosal FISH analysis was available in 3579 of 4366 NIPT-negative cases (81.8%). Based on postnatal cytogenetic confirmation using neonatal buccal mucosal FISH, two FN cases were identified among 3579 NIPT-negative pregnancies (0.06%), resulting in NPV of 99.9% for trisomies 13, 18, and 21.

**Fig. 2:**
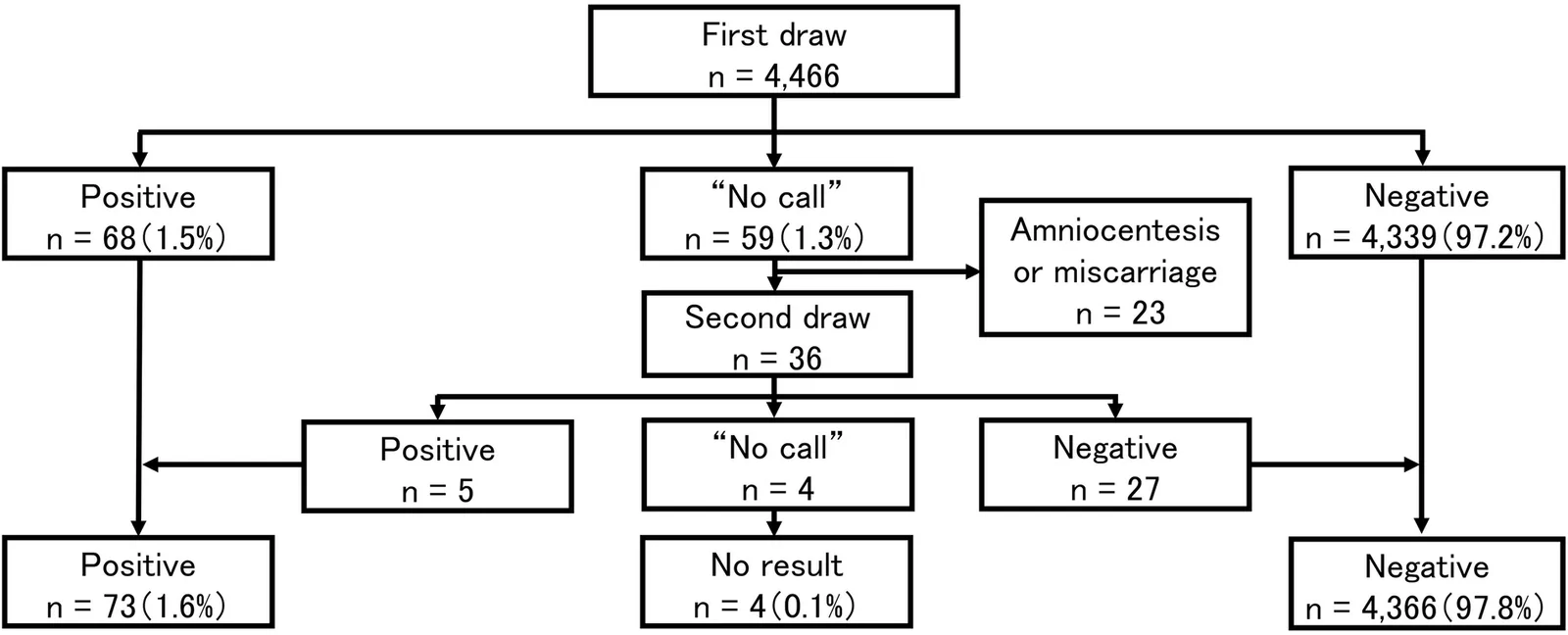
Flowchart of SNP-based NIPT results. Results from the first draw and outcomes after second draw are shown

**Fig. 3:**
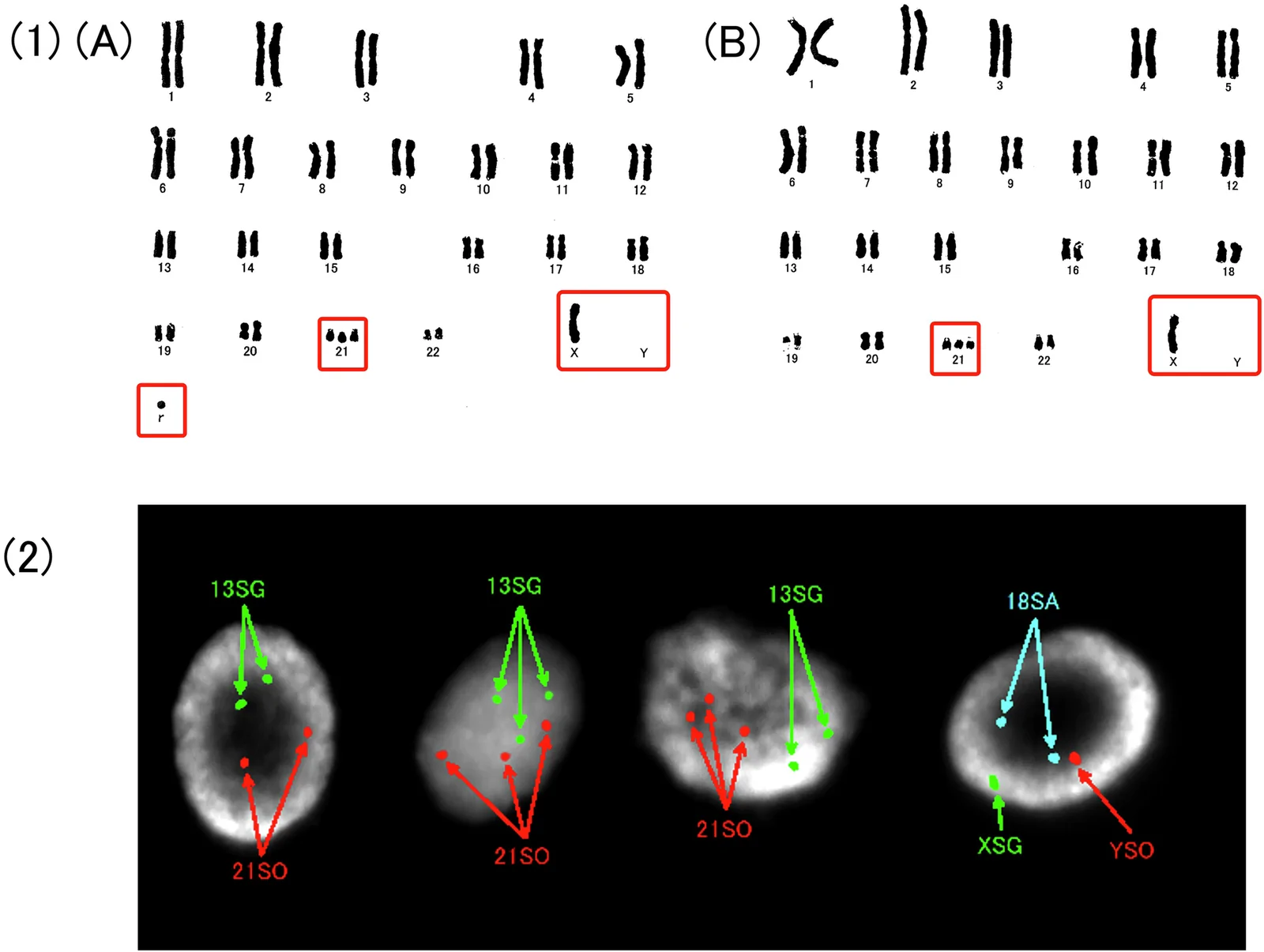
Cytogenetic findings in two false-negative cases. (1) False-negative Case 1: Mosaic trisomy 21 with X monosomy identified by amniocentesis. (**A**) Representative karyotype of the 48,X,+21,+r cell line. (**B**) Representative karyotype of the 46,X,+21 cell line. FISH analysis of amniotic fluid showed mosaic aneuploidy consisting of two cell lines: 48,X,+21,+r[47/51] and 46,X,+21[4/51]. Sex chromosome signals were also mosaic, with 22 cells showing monosomy X and 29 cells showing two sex-chromosome signals. G-banding analysis of 23 cultured cells demonstrated the same mosaic pattern (13 cells 48,X,+21,+r; 10 cells 46,X,+21). (2) False-negative Case 2: Low-frequency mosaic trisomy 21 and trisomy 13 detected by postnatal cytogenetic analysis. Neonatal buccal mucosal FISH analysis following a negative NIPT result demonstrated three cell lines: 46,XY[90/100], 47,XY,+21[7/100], and 48,XY,+13,+21[3/100], indicating low-level mosaicism for trisomy 21 and trisomy 13

**FN Case 1**: A 41-year-old woman conceived naturally and underwent NIPT at 13 weeks due to advanced maternal age. The result was negative; however, marked nuchal translucency thickening (7 mm) was observed on fetal ultrasound, and amniocentesis was performed after genetic counseling. FISH revealed mosaic trisomy 21 with X monosomy (47,X, + 21,+r (7%)/ 46,X, + 21 (93%)), consistent with G-banding findings. After genetic counseling, the couple chose to terminate the pregnancy (Figs. 1–3).

**FN Case 2**: A 31-year-old woman conceived through assisted reproductive technology. NIPT was performed at 12 weeks of gestation due to a history of a chromosomally abnormal pregnancy. The result was negative, and the pregnancy continued without any abnormal findings during pregnancy or at birth. Neonatal buccal mucosal FISH analysis revealed three cell lines:46,XY (90%), 47,XY, + 21 (7%), and 48,XY, + 13, + 21 (3%), indicating low-level mosaicism for trisomy 13 and 21 (Figs. 2, 3).

### Case of “no call” (Table 2)

The frequency of “no call” at the first draw NIPT was 1.3% (59/4466). The reasons for “no call” were as follows: low FFR < 4% in 14 of 59 cases (22.0%), uninterpretable DNA pattern in 35 cases (59.3%), and unrecognized vanishing twin or suspected triploidy in 11 cases (18.6%). Uninterpretable DNA patterns refer to cases in which SNP-based haplotype analysis failed to meet predefined quality-control criteria, including excessive background noise, insufficient informative SNPs, or inability to reliably reconstruct fetal haplotypes, despite adequate sequencing depth. Among those who underwent redraw testing, 88.9% obtained reportable results, and only four cases (0.1% of all tests) remained “no call” (Table 2). One persistent “no call” case was complicated by systemic lupus erythematosus (SLE), and three occurred in women with BMI close to 30. None were receiving anticoagulation therapy. Two patients underwent amniocentesis, both confirming a normal karyotype, whereas the remaining two continued their pregnancies without any abnormal findings by ultrasound. Four cases did not undergo confirmation by neonatal buccal mucosal FISH analysis.

**Table 2:** Clinical characteristics of cases with repeated “no call” results

Case		1	2	3	4
Age		39	38	39	40
BMI		34.2	29.4	36.8	27.8
Complication		no	no	no	SLE
Ultrasound abnormality		no	no	no	no
First draw	Gestational week	12	14	12	13
	FFR(%)	3.5	4.7	3.1	3.7
Second draw	Gestational week	14	16	14	15
	FFR(%)	3	4	2.2	3.7
Amniocentesis		46,XX	46,XY	Not performed	Not performed

*FFR* fetal fraction rate, *SLE* systemic lupus erythematosus

### Sensitivity and specificity (Table 3)

Sensitivity and specificity were calculated based on confirmation by amniocentesis or subsequent neonatal buccal mucosal FISH analysis. FN cases with mosaicism were also classified as aneuploid (positive) and included in the calculations (Table 3). Diagnostic performance was evaluated separately for each chromosome; therefore, cases with multiple chromosomal abnormalities were included in the analysis of each relevant chromosome. The sensitivity for trisomy 13, 18, and 21 was 94.6%, 100.0%, and 80.0%, respectively, and the specificity was 99.9% for all three conditions.

**Table 3:** Sensitivity and specificity of SNP-based NIPT for trisomies 13, 18, and 21

	Sensitivity	95% CI	Specificity	95% CI
Trisomy 21	94.6 (35/37)	82.3–98.5	99.9 (3577/3579)	99.8–99.9
Trisomy 18	100.0 (18/18)	82.3–100.0	99.9 (3579/3581)	99.8–99.9
Trisomy 13	80.0 (4/5)	37.6–96.4	99.9 (3578/3581)	99.8–99.9

*95% CI* 95% confidence interval

## Discussion

This study represents, to our knowledge, the first report in Japan to validate SNP-based NIPT using neonatal buccal mucosal FISH, demonstrating high screening performance (PPV 89.1%, NPV 99.9%). Notably, this single-center study includes one of the largest cohorts reported in Japan, with comprehensive cytogenetic confirmation not only in positive cases but also across a large number of negative cases.

### Validation of the test

The accuracy of SNP-based NIPT was previously evaluated by Dar et al. in a large-scale study published in 2014, which reported a PPV of 82.9% (90.2% for trisomy 21) based on follow-up of 17,885 of 31,030 pregnancies. However, FN identification depended on clinical follow-up and voluntary reporting by healthcare providers, likely leading to underestimation of the true false-negative rate [6]. In our study, the PPV was 89.1% (94.6% for trisomy 21), consistent with these previous findings.

In Japan, MPS-based NIPT has been predominant since NIPT was first introduced in 2013. In large multicenter studies, participating institutions accumulated their results using platform-specific NIPT assays. In a representative study using the MPS method (Genetech®), among 44,263 cases, 59 were FP and three were FN (one trisomy 21 and two trisomy 18). Reported sensitivities for trisomies 21, 18 and 13 were 99.78%, 99.12%, and 100% respectively, and specificities were 99.97%, 99.96%, and 99.94%; and the PPVs at maternal age 35 years were 93%, 77%, and 43% [7].

However, previous study had theoretically and statistically important limitations, including the exclusion of multiple pregnancies and vanishing twins, as well as the classification of cases with unconfirmed results as positive, while negative cases were largely classified based on postnatal phenotypic observation without systematic cytogenetic confirmation.

In contrast to previous studies, in which negative NIPT results were often assessed based on postnatal phenotype alone, the present study incorporated neonatal buccal mucosal FISH analysis for negative NIPT results whenever possible, allowing a more reliable evaluation of analytical performance. However, cytogenetic confirmation was available in 81.8% of NIPT-negative cases and was not complete. Therefore, although our study provides more systematic cytogenetic follow-up than many previous reports, incomplete follow-up may still have resulted in a small underestimation of false-negative cases.

### Causes of FN

In our cohort, two FN cases were identified (2/3579, 0.06%). Previous large-scale studies have reported similarly low FN frequencies. For example, one investigation in a mixed population estimated an FN rate of approximately 1 in 16,000 (0.006%) [12], while another large commercial cohort reported a rate of 1 in 9,000 (0.01%) in a similarly mixed-risk population [6]. Although the mechanisms underlying discordant NIPT results are not fully understood, a review has suggested several potential causes, including placental mosaicism, maternal copy number variation, vanishing twin, maternal malignancy, and true fetal mosaicism [13, 14].

In the present study, both FN cases were attributable to low-level mosaic aneuploidy confirmed after birth. Mosaicism is a well-recognized cause of FN results because cfDNA in maternal plasma originates predominantly from placental trophoblasts. When the abnormal cell line is under-represented in placental tissue relative to fetal tissues, the aneuploid signal may fall below the analytical detection threshold. In addition, discordance between placental and fetal mosaicism is well documented, and the proportion of mosaic cell lines can vary substantially between placental and fetal tissues.

Importantly, these biological mechanisms could be elucidated in our cohort owing to the relatively high rate of postnatal cytogenetic confirmation. Although approximately 89.1% of the study population received prenatal care at external institutions, reflecting the referral-based nature of NIPT practice during the study period, cytogenetic follow-up using neonatal buccal mucosal FISH was achieved in 81.8% of cases. This relatively high follow-up rate may be attributable to the structured follow-up system, including the timely distribution of sampling kits and the simplicity of specimen collection, which facilitated patient compliance even in a decentralized care setting.

Taken together, these findings underscore that the detection of chromosomal mosaicism remains inherently challenging across all NIPT platforms, including both SNP-based and MPS-based approaches. Therefore, discordant results, including false-negatives, may arise not only from technical limitations but also from fundamental biological factors such as confined placental mosaicism and tissue-specific mosaicism.

### “No call” cases

In this study, 1.3% of the cases did not yield results in the initial draw, but 88.9% of redraw tests produced reportable outcomes. According to the report of “no call” results in MPS-based NIPT in Japan, 110 cases (0.32%) out of 34,626 first draw did not yield results; among 94 redraws, 61 (64.9%) yielded results, while 33 (35.1%) remained “no call”, resulting in no call rate of 0.1% [15]. The final “no call” rate in our SNP-based cohort was likewise 0.1%.

Of the four persistent “no call” cases in this study, three occurred in women with BMI around 30, and were associated with low FFR ( ≤ 5%). FFR increases with gestational age [16] but decreases with maternal weight [17]. Therefore, it is highly likely that obesity-related low FFR contributed to repeated test failure.

The remaining case involved a patient with SLE and a low FFR (3.7%). Previous studies have suggested that maternal autoimmune disorders may influence cfDNA characteristics and fetal fraction [18, 19]. For example, biological alterations of circulating DNA have been reported in SLE. Active SLE has been associated with a marked increase in short cfDNA fragments, with fragments ≤115 bp occurring approximately three times more frequently than in healthy individuals and accounting for up to 84% of plasma DNA. Such fragmentation patterns may alter the representation of cfDNA molecules and could potentially affect the performance or interpretation of cfDNA-based prenatal screening tests [19]. However, the majority of data available originates from MPS-based NIPT platforms, while evidence concerning SNP-based NIPT remains limited. It is therefore possible that maternal autoimmune conditions or related treatments may contribute to low fetal fraction and an increased likelihood of “no call”, although further studies are needed to clarify platform-specific effects.

In cases of “no call,” decisions regarding redraw or diagnostic testing should be considered based on maternal characteristics and gestational age.

### Regulatory background of NIPT in Japan

In Japan, the Maternal Protection Law does not contain explicit provisions regarding fetal indications. Consequently, ethical and social discussions surrounding prenatal testing have historically been approached with caution. Within this context, JSOG issued the “Opinion on Genetic Testing and Diagnosis Performed Prenatally,” which established the fundamental principles for prenatal genetic testing in Japan [20].

Based on this framework, NIPT was introduced in Japan in 2013 within a carefully regulated clinical research setting. The present study was also initiated in accordance with this opinion and was approved by the institutional ethics committee of our hospital.

Subsequently, from around 2015, unlicensed facilities of NIPT began to increase, raising concerns regarding the adequacy of genetic counseling provided before and after testing, as well as the follow-up system for cases with positive results. In the present study, the number of NIPT tests peaked in 2015 and subsequently declined. This trend may partly reflect the expansion of NIPT services at unlicensed facilities during this period.

In response to these circumstances, JSOG revised its draft guidelines based on the perspectives of the Japan Pediatric Society and the Japan Society of Human Genetics. Following discussions and consensus among these societies, the revised proposal was submitted to the Ministry of Health, Labor and Welfare. After several deliberations by the Science and Technology Subcommittee of the Health Science Council in 2021, a public report on prenatal testing was issued, which reorganized the national framework for prenatal testing in Japan.

As part of this reform, the approach to providing information on prenatal testing was also reconsidered. The previous practice of explaining NIPT only to individuals who explicitly requested the test was replaced by a policy encouraging healthcare providers to offer information on prenatal testing as part of comprehensive support for pregnancy, childbirth, and child-rearing. This approach aims to ensure that pregnant women and their partners can correctly understand the nature and implications of prenatal testing and make well-considered decisions regarding whether to undergo testing and which test to choose. In the present study, the research design and operational framework were adjusted as appropriate in response to these policy changes, and this study was concluded in 2022.

Within this regulatory context, the present study was conducted under a structured clinical research framework that emphasized appropriate genetic counseling and informed decision-making, in contrast to unlicensed facilities. A multidisciplinary team consisting of obstetricians, clinical geneticists, and certified genetic counselors was involved in the counseling process. When necessary, patients were referred to pediatric genetic specialists for additional counseling. The research team also periodically reviewed the study framework within the institution to ensure compliance with evolving institutional policies and ethical requirements throughout the study period.

Thus, the present study provides real-world clinical data from the early phase of NIPT implementation in Japan, when the test was introduced within a strictly regulated research framework. These findings provide important insights into the real-world performance of SNP-based NIPT and may contribute to the development of prenatal testing policies and clinical practice in Japan.

### Limitations

First, although clinical data were prospectively collected within a clinical research framework, this single-center study with a limited sample size may limit the generalizability of the findings.

Second, neonatal cytogenetic confirmation was performed in 81.8% of cases, whereas it was unavailable in the remaining 18.2%. Although NIPT was performed at our institution, 89.1% of pregnancies were managed and delivered at other institutions, as only 34 certified facilities were available in Japan when NIPT was introduced in 2013, resulting in a large number of referrals from other centers due to limited access. Therefore, systematic postnatal follow-up, including review of pediatric medical records and physical examinations, was not available for cases without neonatal cytogenetic confirmation.

Third, there are limitations related to the reference standard. Ideally, confirmation of NIPT results would rely on analyses using amniotic fluid–derived samples, such as targeted FISH, QF-PCR, or chromosomal microarray analysis using uncultured amniocytes, supplemented by conventional karyotyping after cell culture. However, performing amniocentesis in all cases undergoing NIPT is neither ethically nor practically feasible. Therefore, neonatal buccal mucosal FISH was used in this study as a practical validation method because of its low invasiveness and high feasibility in terms of cost and implementation. However, buccal epithelial cells may not fully reflect chromosomal abnormalities present in other fetal tissues, and discordance with NIPT results may occur due to tissue-specific mosaicism. Consequently, the analytical sensitivity reported in this study should be interpreted with caution, as it may have been influenced not only by methodological limitations of FISH but also by biological discordance between placental and fetal tissues. This limitation is not unique to SNP-based NIPT but is inherent to all current prenatal testing methodologies, including MPS-based approaches.

## Conclusion

In conclusion, SNP-based NIPT demonstrated high analytical performance when validated against invasive prenatal testing and neonatal buccal mucosal FISH analysis. However, rare false-negative results due to low-level mosaic aneuploidy were identified, highlighting the biological limitations of cfDNA-based screening. Therefore, invasive diagnostic testing, such as amniocentesis, should be considered when clinical findings raise concern for chromosomal abnormalities, even after a negative NIPT.
